# Global-scale phylogenetic linguistic inference from lexical resources

**DOI:** 10.1038/sdata.2018.189

**Published:** 2018-10-09

**Authors:** Gerhard Jäger

**Affiliations:** 1 Tübingen University, Institute of Linguistics, Wilhelmstr. 19, 72074 Tübingen, Germany

**Keywords:** Phylogeny, Statistical methods

## Abstract

Automatic phylogenetic inference plays an increasingly important role in computational historical linguistics. Most pertinent work is currently based on *expert cognate judgments*. This limits the scope of this approach to a small number of well-studied language families. We used machine learning techniques to compile data suitable for phylogenetic inference from the ASJP database, a collection of almost 7,000 phonetically transcribed word lists over 40 concepts, covering two thirds of the extant world-wide linguistic diversity. First, we estimated *Pointwise Mutual Information* scores between sound classes using weighted sequence alignment and general-purpose optimization. From this we computed a dissimilarity matrix over all ASJP word lists. This matrix is suitable for *distance-based* phylogenetic inference. Second, we applied *cognate clustering* to the ASJP data, using supervised training of an SVM classifier on expert cognacy judgments. Third, we defined two types of binary *characters*, based on automatically inferred cognate classes and on sound-class occurrences. Several tests are reported demonstrating the suitability of these characters for *character-based* phylogenetic inference.

## Background & Summary

The cultural transmission of natural languages with its patterns of near-faithful replication from generation to generation, and the diversification resulting from population splits, are known to display striking similarities to biological evolution^1,2^. The mathematical tools to recover evolutionary history developed in computational biology — phylogenetic inference — play an increasingly important role in the study of the diversity and history of human languages^3–14^.

The main bottleneck for this research program is the presently limited availability of suitable data. Most extant studies rely on manually curated collections of expert judgments pertaining to the cognacy of core vocabulary items or the grammatical classification of languages. Collecting such data is highly labor intensive. Therefore sizeable collections currently exist only for a relatively small number of well-studied language families^8,11,15–18^.

Basing phylogenetic inference on expert judgments, especially judgments regarding the cognacy between words, also raises methodological concerns. The experts making those judgments are necessarily historical linguists with some prior information about the genetic relationships between the languages involved. In fact, it is virtually impossible to pass a judgment about cognacy without forming a hypothesis about such relations. In this way, data are enriched with prior assumptions of human experts in a way that is hard to control or to precisely replicate.

Modern machine learning techniques provide a way to greatly expand the empirical base of phylogenetic linguistics while avoiding the above-mentioned methodological problem.

The *Automated Similarity Judgment Program* (ASJP; see Data Citation 1) database contains 40-item core vocabulary lists from more than 7,000 languages and dialects across the globe, covering about 75% of the extant linguistic diversity. All data are in phonetic transcription with little additional annotations. (The only expert judgments contained in the ASJP data are rather unsystematic manual identifications of loan words. This information is ignored in the present study). It is, at the current time, the most comprehensive collection of word lists available.

Phylogenetic inference techniques comes in two flavors, *distance-based* and *character-based* methods. Distance-based methods require as input a matrix of pairwise distances between taxa. Character-based methods operate on a character matrix, i.e. a classification of the taxa under consideration according to a list of discrete, finite-valued characters. Character-based methods more directly infer the evolutionary process of descent with modification, but they can be computationally expensive. Distance-based methods return a tree diagram which groups taxa according to similarity, which does not necessarily equal relatedness (shared characteristics through common descent) but is highly computationally efficient and can be adequate for some purposes or useful as a first approximation.

The literature contains proposals to extract both pairwise distance matrices and character data from phonetically transcribed word lists^19–21^. In this paper we apply those methods to the ASJP data and make both a distance matrix and a character matrix for 6,892 languages and dialects—these are all languages in ASJP v. 17 except reconstructed, artificial, pidgin and creole languages—derived this way available to the community. Also, we demonstrate the suitability of the results for phylogenetic inference. The results of phylogenetic inference, i.e., fully bifurcating phylogenetic trees including branch lengths, for 66 language families and for the entire set of 6,892 of languages and dialects are also made publicly available. While these trees still await a detailed qualitative assessment by trained comparative linguists, they are (by construction) compatible with the Glottolog (see Data Citation 2) classification. This provides a useful resource for applications of the *Phylogenetic Comparative Methods* (see, e.g.^22^, for an overview) to questions in linguistic typology.

While both the raw data and the algorithmic methods used in this study are freely publicly available, the computational effort required was considerable (about ten days computing time on a 160-cores parallel server). Therefore the resulting resource is worth publishing in its own right.

## Methods

### Creating a distance matrix from word lists

In^19^ a method is developed to estimate the dissimilarity between two ASJP word lists. The main steps will be briefly recapitulated here.

#### Pointwise Mutual Information

ASJP entries are transcribed in a simple phonetic alphabet consisting of 41 sound classes and diacritics. (See^6^ for a detailed description of the code). In all steps described in this paper, diacritics are removed. For instance, a sequence th, indicating an aspirated “t”, is replaced by a simple t. This way, each word is represented as a sequence over the 41 ASJP sound classes.

The *pointwise mutual information* (PMI) between two sound classes is central for most methods used in this paper. It is defined as

(1)PMI(a,b)≐−logs(a,b)q(a)q(b),

where *s*(*a*, *b*) is the probability of an occurrence of *a* to be cognate with *b* in a pair of cognate words, and *q*(*x*) are the probabilities of occurrence of *x* in an arbitrarily chosen word.

Let “-” be the gap symbol. A pairwise alignment between two strings (*x*,*y*) is a pair of strings (x′,y′) over sound class symbols and gaps of equal length such that *x* is the result of removing all gap occurrences in *x*′, and likewise for y′. A licit alignment is one where a gap in one string is never followed by a gap in the other string. There are two parameters *gp*_1_ and *gp*_2_, the *gap penalties* for opening and extending a gap. The aggregate PMI of an alignment is

(2)PMI(xi',yi')=∑iPMI(xi',yi'),

where $PMI(x_{i}^{'},y_{i}^{'})$ is the corresponding gap penalty if $x_{i}^{'}$ or $y_{i}^{'}$ is a gap.

For a given pair of ungapped strings (*x*, *y*), *PMI*(*x*, *y*) is the maximal aggregate PMI of all possible licit alignments between *x* and *y*. It can efficiently be computed with a version of the Needleman-Wunsch algorithm^23^. In this study, we used the function pairwise2.align.globalds of the *Biopython* library^24^ for performing alignments and computing PMI scores between strings.

### Parameter estimation

The probabilities of occurrence *q*(*a*) for sound classes *a* are estimated as relative frequencies of occurrence within the ASJP entries. The scores *PMI*(*a*,*b*) for pairs of sound classes (*a*,*b*) and the gap penalties are estimated via an iterative procedure.

In a first step, pairwise distances between languages are computed via the method described in the next subsection, using 1-*LDN*(*x*, *y*) instead of *PMI*(*x*, *y*) as measure of string similarity, where *LDN*(*x*, *y*) is the *normalized Levenshtein distance*^25^ between *x* and *y*, i.e. the edit distance between *x* and *y* divided by the length of the longest string. In the next subsection I will describe a method how distances between strings can be aggregated to yield a distance measures between languages (i.e., word lists). (For the sake of readability, I will use the term “language” to refer to languages proper and to dialects alike; “doculect” would be a more correct if cumbersome term. A *doculect* is any linguistic variety (language, dialect, sociolect etc.) that “has been described or otherwise documented in a coherent way” (http://www.glottopedia.org/index.php/Doculect, accessed on June 12, 2018.) Working with doculects allows us to remain neutral about the notoriously difficult language/dialect distinction.). All pairs of languages (*l*_1_*l*_2_) where this distance ≤0.7 are considered as *probably related*. This threshold was chosen somewhat arbitrarily but is highly conservative; 99.9% of all probably related languages belong to the same language family and about 60% to the same sub-family.

Next, for each pair of probably related languages (*l*_1_, *l*_2_) and each concept *c*, each word for *c* from *l*_1_ is aligned to each word for *c* from *l*_2_. The pair of words with the lowest *LDN* score is considered as *potentially cognate*.

All pairs of potentially cognate words are aligned using the Levenshtein algorithm, and for each pair of sound classes (*a*, *b*), *s*_0_(*a*, *b*) is estimated as the relative frequency of *a* being aligned to *b* across all such alignments. Alignments to gaps are excluded from this computation. *PMI*_0_(*a*, *b*) is then calculated according to (1). As pointed out by a reviewer, this procedure gives more weight to large families, as there are much more pairs of probably related languages from large families than from small ones. Under the quite plausible prior assumption, however, that the probability of sound changes is not lineage-dependent, this does not lead to biased estimates though.

Suppose gap penalties *gp*_1_, *gp*_2_ and a threshold parameter *θ* are given. The final PMI scores are estimated using an iterative procedure inspired by the *Expectation Maximization* algorithm^26^:

- For *i* in 1…10:
- All potential cognate pairs are aligned using the *PMI*_*i*-1_-scores.
- *s*_*i*_(*a*, *b*) is estimated as the relative frequency of *a* aligned with *b* among all alignments between potential cognates *x*,*y* with *PMI*_*i*-1_(*x*, *y*)≥*θ*.
- *PMI*_*i*_ is calculated using formula (1).

The *target function f*(*gp*_1_, *gp*_2,_ *θ*) is the average distance between all probably related languages using the *PMI*_10_-scores. The values for *gp*_1,_ *gp*_2,_ *θ* are determined as those minimizing *f*, using Nelder-Mead optimization^27^. The following optimal values were found: $gp_{1}\approx -2.330,gp_{2}\approx -1.276,\theta \approx 4.401$.

The threshold *θ* ≈ 4.401 ensures that only highly similar word pairs are used for estimating PMI scores. For instance, between French and Italian only five word pairs have a PMI similarity ≥*θ* according to the final scores: *soleil*
[sole] - *sole*
[sole] (‘sun’; PMI = 11.6), *corne*
[korn] - *corno*
[korno] (‘horn’; PMI = 7.7), *arbre*
[arbr3] - *albero*
[albero] (‘tree’; PMI = 7.1), *nouveau*
[nuvo] - *nuovo*
[nwovo] (‘new’; PMI = 7.0), and *montagne*
[motaj] - *montagna*
[monta5a] (‘mountain’; PMI = 4.9).

**Figure 1:**
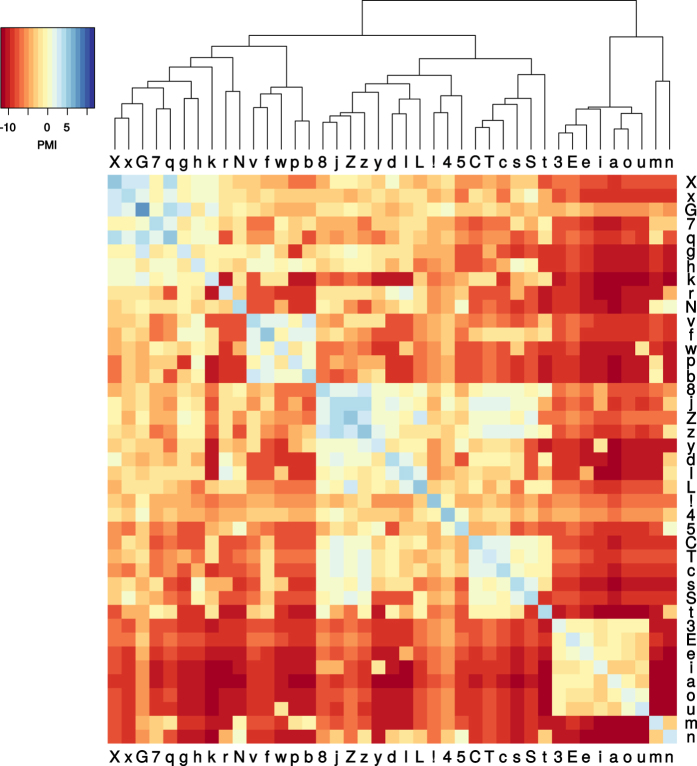
PMI scores. Heatmap and hierarchical clustering dendrogram.

The final PMI scores between sound classes are visualized in Fig. 1. It is easy to discern that *PMI*(*a*,*a*) is positive for all sound classes *a*, and that *PMI*(*a*,*b*) for *a* ≠ *b* is negative in most cases. There are a few pairs *a,b* with positive score, such as b/f. Generally, sound class pairs with a similar place of articulation tend to have relatively high scores. This pattern is also visible in the cluster dendrogram. We observe a primary split between vowels and consonants. Consonants are further divided into labials, dentals, and velar/uvular sounds.

It should be noted that the pairwise PMI-scores between sound classes measure something like the propensity of these sound classes to participate in a sound correspondence; it does not say anything about sound correspondences between specific pairs of languages. The ASJP word lists, with just 40 items, are definitely too short to infer sound correspondences between individual language pairs.

### Pairwise distances between languages

When aggregating PMI similarities between individual words into a distance measure between word lists, various complicating factors have to be taken into consideration:

- Entries for a certain language and a certain concept often contain several synonyms. This is a potential source of bias when averaging PMI similarities of individual word pairs. (As correctly pointed out by a reviewer, this effect is aggrevated by the observation that the number of synonyms may reflect the extent and quality of the language’s documentation. Currently I see no way to control for this kind of bias).
- Cognate words tend to be more similar than non-cognate ones. However, the average similarity level between non-cognate words depends on the overall similarity between the sound inventories and phonotactic structure of the languages compared. To assess the informativeness of a certain PMI similarity score, it has to be calibrated against the overall distribution of PMI similarities between non-cognate words from the languages in question.
- Many ASJP word lists are incomplete, so the word lists are of unequal length.

To address the first problem, ^19^ defined the similarity score between languages *l*_1_ and *l*_2_ for concept *c* as the maximal PMI similarity between any pair of entries for *c* from *l*_1_ and *l*_2_.

The second problem is addressed by estimating, for each concept *c* for which both languages have an entry, the *p*-value for the null hypothesis that none of the words for *c* being compared are cognate. This is done in a parameter-free way. For each pair of concepts (*c*_1_,*c*_2_), the PMI similarities between the words for *c*_1_ from *l*_1_ and the words for *c*_2_ from *l*_2_ are computed. The maximum of these values is the similarity score for (*c*_1_,*c*_2_). Under the simplifying assumption that cognate words always share their meaning the distribution of such similarity scores for *c*_1_≠*c*_2_ constitutes a sample of the overall distribution of similarity scores between non-cognates.

It should be noted that the assumption of general synomymity of cognates is evidently false when considering the entire lexicon. There is a plethora of examples, such as as English *deer* vs. German *Tier* ‘animal’, which are cognate (cf.^28^, p. 94) without being synonyms. However, within the 40-concept core vocabulary space covered by ASJP, such cross-concept cognate pairs are arguably very rare.

Now consider the null hypothesis that the words for concept *c* are non-cognate. We assume *a priori* that cognate word pairs are more similar than non-cognate ones. Let the similarity score for *c* be *x*. The maximum likelihood estimate for the *p*-value of that null hypothesis is the relative frequency non-cognate pairs with a similarity score ≥*x*. If *PMI*(*c*_*i*_,*c*_*j*_) is the similarity &score between concept *c*_*i*_ and *c*_*j*_, we have

(3)pc=|{(c, c)} ∪{(ci,cj)|ci≠cj&PMI(ci,cj)≥PMI(c,c)||{(c,c)}∪{(ci,cj)|ci≠cj}|.

Analogously to Fisher’s method^29^, the *p*-values for all concepts are combined according to the formula

(4)∑c−logpc.

If the null hypothesis is true for concept *c*, *p*_*c*_ is distributed approximately according to a continuous uniform distribution over the interval (0, 1]. Accordingly, –log *p*_*c*_ is distributed according to an exponential distribution with mean and variance =1.

Suppose there are *N* concepts for which both *l*_1_ and *l*_2_ have an entry. The sum of *N* independently distributed random variables, each with mean and variance =1, approximately follows a normal distribution with mean =*N* and variance =*N*. This can be transformed into a *Z*-statistic by normalizing according to the formula

(5)Z(l1,l2)=∑i=1N−logpci−NN

This normalization step addresses the third issue mentioned above, i.e., the varying length of word lists.

*Z*(*l*_1_, *l*_2_) increases with the degree of similarity between *l*_1_ and *l*_2_. It is transformed into a dissimilarity measure as follows: (We will talk of *distance measure* from now on for simplicity, even though it is not a metric distance).

(6)d(l1,l2)=Zmax−Z(l1,l2)Zmax−Zmin

The maximal possible value *Z*_mix_ for *Z* would be achieved if both word lists have the maximal length of *N*=40, and each synonymous word pair has a higher PMI score than any non-synonymous word pair. Therefore

$$Z_{\max}=\frac{40\times -\log\frac{1}{40^{2}-40+1}-40}{\sqrt{40}}\approx 40.18$$

The minimal value *Z*_min_ for *Z* would be achieved if all *p*_*c*_ equal 1 and both word lists have length 40:

$$Z_{\min}=\frac{40\times -\log1-40}{\sqrt{40}}=-\sqrt{40}\approx -6.32$$

We computed *d*(*l*_1_, *l*_2_) for each pair of the above-mentioned 6,892 languages from the ASJP database. This distance matrix is available at (Data Citation 3).

### Automatic cognate classification

#### Background

In^20^ a method is developed to cluster words into equivalence classes in a way that approximates manual expert classifications. In this section this approach is briefly sketched.

The authors chose a supervised learning approach. They use word lists with manual expert cognate annotations from a diverse collection of language families, taken from^15–18^; http://ielex.mpi.nl. A part of these gold standard data were used to train a *Support Vector Machine* (SVM). For each pair of words (*w*_1,_*w*_2_) from languages (*l*_1_, *l*_2_), denoting concept *c*, seven feature values were computed:

1. **PMI similarity.** This is the string similarity measure according to^19^ as described in the previous section.
2. **Calibrated PMI distance.** *p*_*c*_ as defined in equation (3) above.
3. The negative logarithm thereof.
4. **Language similarity.** Z(*l*_1_, *l*_2_), as defined in equation (5) above.
5. The logarithm thereof.
6. **Average word length** of words for concept *c* across all languages from the database, measured in number of symbols in ASJP transcription.
7. **Concept-language correlation.** The Pearson correlation coefficient between feature 3 and feature 4 for all word pairs expressing concept *c*.

For each such word pair, the gold standard contains an evaluation as *cognate* (1) or *not cognate* (0). An SVM was trained to predict these binary cognacy labels. Applying Platt scaling^30^, the algorithm predics a *probability of cognacy* for each pair of words from different languages denoting the same concept. These probabilities were used as input for hierarchical clustering, yielding a partitioning of words into equivalence classes for each concept.

The authors divided the gold standard data into a training set and a test set. Using an SVM trained with the training set, they achieve B-cubed F-scores^31^ between 66.9% and 90.9% on the data sets in their test data, with a weighted average of 71.8% when comparing automatically inferred clusters with manual cognate classifications. (The *F-score* is an aggregate measure relying both on the *precision* — the proportion of predicted cognates that actually are cognate — and the *recall* — the proportion of true cognates that are correctly identified).

In^20^ it is shown that this approach leads to slightly improved results if compared with LexStat^32^, which can be considered as state of the art. In^21^ it is furthermore demonstrated that the SVM-based approach is especially superior when applied to short and poorly transcribed word lists, while the differences virtually level out for longer and high-quality lists.

### Creating a gold standard

We adapted this approach to the task of performing automatic cognate classification on the ASJP data. Since ASJP contains data from different families and it is confined to 40 core concepts (while the data used in^20^ partially cover 200-item concept lists), the method has to be modified accordingly.

We created a gold standard dataset from the data used in^21^ (which is is drawn from the same sources as the data used in^20^ but has been manually edited to correct annotation mistakes). Only the 40 ASJP concepts were used. Also, we selected the source data in such a way that each dataset is drawn from a different language family. Words from different families were generally classified as non-cognate in the gold standard. All transcriptions were converted into ASJP format. Table 1 summarizes the composition of the gold standard data.

### Clustering

We used the *Label Propagation* algorithm^33^ for clustering. For each concept, a network is constructed from the words for that concept. Two nodes are connected if and only if their predicted probability of cognacy is ≥0.25. This threshold was chosen somewhat arbitrarily, based on manual trial and error. *Label Propagation* detects community structures within the network, i.e., it partitions the nodes into clusters.

### Model selection

To identify the set of features suitable for clustering the ASJP data, we performed *cross-validation* on the gold standard data. The data were split into a *training set*, consisting of the data from six randomly chosen language families, and a *test set*, consisting of the remaining data. We slightly deviated from^20^ by replacing features 4 and 5 by *language distance d*(*l*_1_, *l*_2_) as defined in equation (6), and –log(1−*d*(*l*_1_, *l*_2_)). Both are linear transformations of the original features and therefore do not affect the automatic classification.

For each of the 127 non-empty subsets of the seven features, an SVM with an RBF-kernel was trained with 7,000 randomly chosen synonymous word pairs from the training set. Explorative tests revealed that accuracy of prediction does not increase if more training data are being used. The trained SVM plus Platt scaling were used to predict the probability of cognacy for each synonymous word pair from the test set, and the resulting probabilities were used for Label Propagation clustering. This procedure was repeated ten times for random splits of the gold standard data into a training set and a test set.

For each feature combination, the B-cubed F-score, averaged over the ten training/test splits, was determined. The best performance (average B-cubed F-score: 0.86) was achieved using just two features:

- **Word similarity.** The negative logarithm of the calibrated PMI distance, and
- **Language log-distance.** –log(1–*d*(*l*_1_, *l*_2_)), with *d*(*l*_1_, *l*_2_) as defined in equation (6).

**Figure 2:**
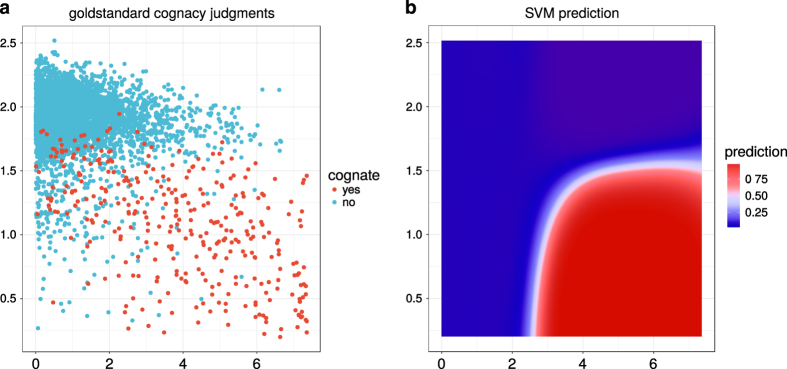
Goldstandard vs. automatic classification. Expert cognacy judgments (**a**) and prediction of cognacy (**b**) depending on the selected features.

Figure 2 displays, for a sample of gold standard data, how expert cognacy judgments depend on these features and how the trained SVM + Platt scaling predicts cognacy depending on those features. Most cognate pairs are concentrated in the lower right corner of the feature space, i.e., they display both high word similarity and low language log-distance. The SVM learns this non-linear dependency between the two features.

### Clustering the ASJP data

A randomly selected sample of 7,000 synonymous word pairs from the gold standard data were used to train an SVM with an RBF-kernel, using the two features obtained via model selection. Probabilities of cognacy for all pairs of synonymous pairs of ASJP entries were obtained by (a) computing word similarity and language log-distance, (b) predict their probability of cognacy using the trained SVM and Platt scaling, and (b) apply Label Propagation clustering.

### Phylogenetic inference

#### Distance-based

The language distances according to the definition in equation (6) can be used as input for distance-based phylogenetic inference. In the experiments reported below, we used the BIONJ^34^ algorithm for that purpose.

#### Character-based

We propose two methods to extract discrete character matrices from the ASJP data.

1. **Automatically inferred cognate classes.** We defined one character per automatically inferred (in the sense described above) cognate class *cc*. If the word list for a language *l* has a missing entry for the concept the elements of *cc* refer to, the character is undefined for this language. Otherwise *l* assumes value 1 if its word list contains an element of *cc*, and 0 otherwise.
2. **Soundclass-concept characters.** We define a character for each pair (*c*,*s*), where *c* is a concept *c* and *s* an ASJP sound class. The character for (*c*,*s*) is undefined for language *l* if *l*'s word list has a missing entry for concept *c*. Otherwise *l*'s value is 1 if one of the words for *c* in *l* contains symbol *s* in its transcription, and 0 otherwise.

The motivation for these two types of characters is that they track two different aspects of language change. Cognacy characters contain information about lexical changes, while soundclass-concept characters also track sound changes within cognate words. Both dimensions provide information about language diversification.

Let us illustrate this with two examples.

- The Old English word for ‘dog’ was *hund*, i.e., hund in ASJP transcription. It belongs to the automatically inferred cognate class *dog_149*. The Modern English word for that concept is *dog*/dag, which belongs to class *dog_150*. This amounts to two mutations of cognate-class characters between Old English and Modern English, 0→1 for *dog_150* and 1→0 for *dog_149*.
- The same historic process is also tracked by the sound-concept characters; it corresponds to five mutations: 0→1 for *dog:a* and *dog:g*, and 1→0 for *dog:h*, *dog:u*, and *dog:n*.
- The word for ‘tree’ changed from Old English *treow*
(treow) to Modern English *tree*
(tri). Both entries belong to cognate class *tree_17*. As no lexical replacement took place for this concept, there is no mutation of cognate-class characters separating Old and Modern English here. The historical sound change processes that are reflected in these words are captured by mutations of sound-concept characters: 0→1 for *tree:i* and 1→0 for *tree:e*, *tree:o* and *tree:w*.

For a given sample of languages, we use all *variable* characters (i.e., characters that have value 1 and value 0 for at least one language in the sample) from both sets of characters. Phylogenetic inference was performed as Maximum-Likelihood estimation assuming Γ-distributed rates with 25 rate categories, and using ascertainment bias correction according to^35^. Base frequencies and variance of rate variation were estimated from the data.

In our phylogenetic experiments, the distance-based tree was used as initial tree for tree search. This method was applied to three character matrices:

- cognate class characters,
- soundclass-concept characters, and
- a partitioned analysis using both types of characters simultaneously.

Inference was performed using the software RAxML^36^.

Applying more advanced methods of character-based inference, such as Bayesian inference^37–39^ proved to be impractical due to hardware limitations.

### Code availability

The code used to conduct this study is freely available at (Data Citation 3). The workflow processes the sub-directories in the following order: 1. pmiPipeline, 2. cognateClustering, and 3. validation. All further details, including software and software versions used, are described in the README files in the individual sub-directories and sub-sub-directories.

## Data Records

All data that were produced are available at (Data Citation 3) as well.

### Phylogenetic trees

Accessible from subdirectory trees/ of (Data Citation 3).

- a family for each of the 66 Glottolog families comprising at least 10 languages in ASJP; the trees were inferred using Maximum Likelihood with combined characters (see Technical Validation for details), using the Glottolog classification as constraint tree. Rooting was performed as described in subsection **A case study: punctuated language evolution**.
- a tree over all 6,982 ASJP languages (world.tre), using the same inference methods, but applying midpoint rerooting.

### PMI data

Accessible from subdirectory pmiPipeline/ of (Data Citation d3).

- estimated PMI scores (pmiScores.csv/) and gap penalties (gapPenalties.csv)
- pairwise distances between languages (pmiWorld.csv)

### Automatic cognate classification

Accessible from subdirectory cognateClustering/ of (Data Citation 3).

• word list with automatically inferred cognate class labels (asjp17Clustered.csv)

### Phylogenetic inference

Accessible from subdirectory validation/ of (Data Citation 3).

- family-wise data and trees (described in Subsection *Phylogenetic Inference* within the Section *Technical Validation*) are in sub-directory validation/ families/. For each Glottolog family F, there are the following files (replace [F] by name of the family):

1. [F].cc.phy: character matrix, cognate class characters, Phylip format
2. [F].sc.phy: character matrix, soundclass-concept characters, Phylip format
3. [F].cc_sc.phy: combined character matrix, cognate class and soundclass-concept characters, Phylip format
4. [F].part.txt: partition file
5. [F].pmi.nex: pairwise PMI distances, Nexus format
6. [F].pmi.tre:BIONJ tree, inferred from PMI distances, Newick format
7. glot.[F].tre: Glottolog tree, Newick format
8. RAxML_bestTree.[F]_cc: Maximum Likelihood tree, inferred from cognate class characters, Newick format
9. RAxML_bestTree.[F]_sc: Maximum Likelihood tree, inferred from soundclass-concept characters, Newick format
10. RAxML_bestTree.[F]_cc_sc: Maximum Likelihood tree, inferred from combined character matrix, Newick format

• global data over all 6,892 languages in the database are in the sub-directory validation/, and global trees in the sub-directory validation/worldTree/:

1. validation/world_cc.phy: character matrix, cognate class characters, Phylip format
2. validation/world_sc.phy: character matrix, soundclass-concept characters, Phylip format
3. validation/world_sc_cc.phy: combined character matrix, cognate class and soundclass-concept characters, Phylip format
4. validation/world.partition.txt: partition file
5. validation/glottologTree.tre: Glottolog tree, Newick format
6. validation/worldTree/distanceTree.tre: BIONJ tree, inferred from PMI distances, Newick format
7. validation/worldTree/RAxML_bestTree.world_cc: Maximum Likelihood tree, inferred from cognate class characters, Newick format
8. validation/worldTree/RAxML_bestTree.world_sc: Maximum Likelihood tree, inferred from soundclass-concept characters, Newick format
9. validation/worldTree/RAxML_bestTree.world_sc_cc: Maximum Likelihood tree, inferred from combined character matrix, Newick format
10. validation/worldTree/RAxML_bestTree.world_sc_ccGlot: Maximum Likelihood tree, inferred from combined character matrix using the Glottolog classifcation as constraint tree, Newick format

For language names, I generally follow the convention [WALS family].[WALS genus].[ASJP doculect name], all in upper case. English, e.g., is named IE.GERMANIC.ENGLISH. If necessary, the corresponding iso codes and other meta-data can easily be accessed from the original ASJP database.

## Technical Validation

### Phylogenetic inference

To evaluate the usefulness of the distance measure and the character matrices defined above for phylogenetic inference, we performed two experiments:

- **Experiment 1**. We applied both distance-based inference and character-based inference for all language families (according to the Glottolog classification) containing at least 10 languages in ASJP.
- **Experiment 2**. We sampled 100 sets of languages with a size between 20 and 400 at random and applied all four methods of phylogenetic inference to each of them.

In both experiments, each automatically inferred phylogeny was evaluated by computing the *Generalized Quartet Distance* (GQD)^40^ to the Glottolog expert tree (restricted to the same set of languages). The GQD gives the proportion quartets of languages that are grouped differently by the expert tree and the automatically generated tree. Quartets that are not *resolved* in the expert tree (e.g., because they come from four different families) are not counted.

**Figure 3:**
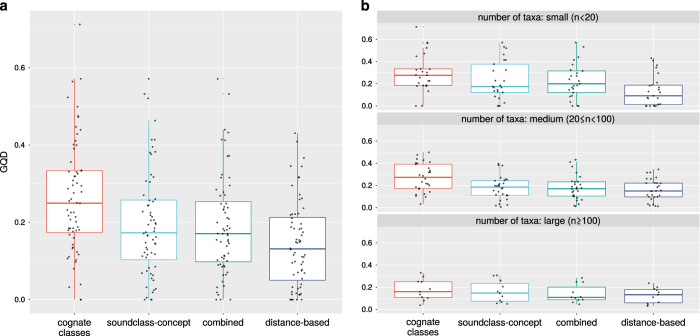
Experiment 1 computed Generalized Quartet Distances for Glottolog families depending on phylogenetic inference method. Aggregated over all families (**a**) and split according to family size (**b**).

The results of the first experiment are summarized in Table 2 and visualized in Fig. 3. The results for the individual families are given in Table 3 (available online only).

Aggregating over all families suggests that distance-based inference produces the best fit with the expert gold standard. However, a closer inspection of the results reveals that the performance of the different phylogenetic inference methods depend on the size of the language families (measured in number of taxa available in ASJP). Combining both types of characters in a partitioned model always leads to better results than the two character types individually. While distance-based inference is superior for small language families (less than 20 taxa), character-based inference appears to be about equally good for medium-sized (20–199 taxa) and large (more than 200 taxa) language families.

**Figure 4:**
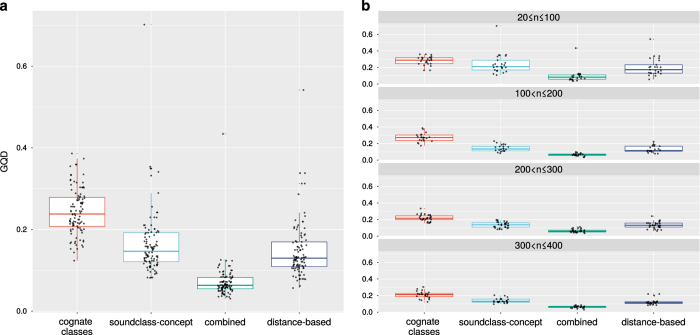
Experiment 2 computed Generalized Quartet Distances for random samples of languages depending on phylogenetic inference method. Aggregated over all samples (**a**) and split according to sample size (**b**).

This assessment is based on a small sample size since there are only 33 medium-sized and 6 large language families. The results of experiment 2 confirm these conclusions though. They are summarized in Table 4 and illustrated in Fig. 4.

All four methods improve with growing sample size, but this effect is more pronounced for character-based inference. While combined character-based inference and distance-based inference are comparable in performance for smaller samples of languages (*n*≤100), character-based inference outperforms distance-based inference for larger samples, and the difference grows with sample size.

The same pattern is found when the different versions of phylogenetic inference is applied to the full dataset of 6,892 languages. We find the following GQD values:

- distance based tree: 0.078
- cognate-class based ML tree: 0.052
- soundclass-concept based ML tree: 0.089
- ML tree from combined character data: 0.035

### Relation to geography

Both the distances between languages and the two methods to represent languages as character vectors are designed to identify similarities between word lists. There are essentially three conceivable causal reasons why the word lists from two languages are similar: (1) common descent, (2) language contact and (3) universal tendencies in sound-meaning association due to sound symbolism, nursery forms etc.^41^. The third effect is arguably rather weak. The signal derived from common descent and from language contact should be correlated with geographic distance. If the methods proposed here extract a genuine signal from word lists, we thus expect to find such a correlation.

To test this hypothesis, we computed the geographic distance (great-circle distance) between all pairs from a sample of 500 randomly selected languages, using the geographic coordinates supplied with the ASJP data.

We furthermore extracted pairwise distances from character vectors by computing the cosine distance between those vectors, using only characters for which both languages have a defined value. In this way we obtained three matrices of pairwise linguistic distances for the mentioned sample of 500 languages: (1) The distance as defined in equation (6), called **PMI distance**, (2) the cosine distance between the cognate-class vectors, and (3) the cosine distance between the sound-concept vectors.

**Figure 5:**
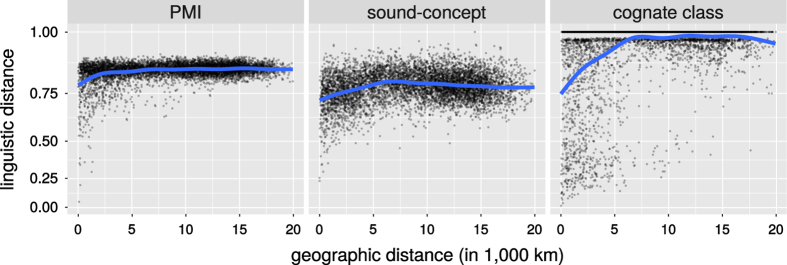
Geographic vs. linguistic distances between languages.

All three linguistic distance measures show a significant correlation with geographic distance. The Spearman correlation coefficients are 0.193 for PMI distances, 0.280 for cognate-class distance and 0.0888 for sound-concept distance. Figure 5 shows the corresponding scatter. The *p*-values according to the Mantel test are ≤0.0001 in all three cases. Figure 5 shows the corresponding scatter plots.

**Figure 6:**
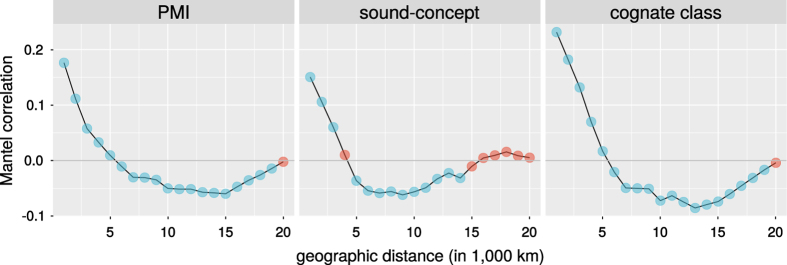
Mantel correlograms. Blue: significant, red: non-significant at *p*<0.05.

The visualization suggests that for all three linguistic distance measures, we find a signal at least up to 5,000 km. This is confirmed by the Mantel correlograms^42^ shown in Fig. 6. We find a significant positive correlation with geographic distance for up to 5,000 km for PMI distance, and up to 4,000 km for cognate-class distance and sound-concept distance.

## Usage Notes

Character-based inference from expert cognacy judgment data have been used in various downstream applications beyond phylogenetic inference, such as estimating the time course of prehistoric population events^3,7,9^ or the identification of overarching patterns of cultural language evolution^5,43^. In this section it will be illustrated how the automatically inferred characters described above can be deployed to expand the scope of such investigations to larger collections of language families. It also demonstrates that automatically inferred branch lengths of phylogenetic trees — a kind of information for which we do not have any manually collected data — provides useful information about language history.

### A case study: punctuated language evolution

A few decades ago, ^44^ proposed that biological evolution is not, in general, a gradual process. Rather, they propose, long periods of stasis are separated by short periods of rapid change co-occurring with branching speciation. This model goes by the name of *punctuated equilibrium*. This proposal has initiated a lively and still ongoing discussion in biology. Pagel, Venditti and Meade^45^ developed a method to test a version of this hypothesis statistically. They argue that most evolutionary change occurs during speciation events. Accordingly, we expect a positive correlation between the number of speciation events a lineage underwent throughout its evolutionary history and the amount of evolutionary change that happened during that time.

Estimates of both quantities can be read off a phylogenetic tree — the number of speciation events corresponds to the number of branching nodes, and the amount of change to the total path length — provided (a) the tree is rooted and (b) branch lengths reflect evolutionary change (e.g., the expected number of mutations of a character) rather than historical time. In^45^ a significant correlation is found for biomolecular data, providing evidence for punctual evolution.

In^43^, the same method is applied to the study of language evolution, using expert cognacy data from three language families (Austronesian, Bantu, Indo-European). The study results in strong evidence for punctuated evolution in all three families.

We conducted a similar study for all Glottolog language families with at least 10 ASJP languages. The workflow was as follows. For each family *F*:

- Find the language *o∉F* which has the minimal average PMI distance to the languages in *F*. This language will be used as *outgroup*.
- Infer a Maximum-Likelihood tree over the taxa *F*∪{*o*} with the Glottolog classification as constraint tree, using a partitioned analysis with cognate-class characters and soundclass-concept characters.
- Use *o* as outgroup to root the tree; remove *o* from the tree.
- Apply the *δ-test*^46^ to control for the *node density artifact*.
- Perform *Phylogenetic Generalized Least Square*^47^ regression with root-to-tip path lengths for all taxa as independent and root-to-tip number of nodes as dependent variable.
- If the *δ*-test is negative and the regression results in a significantly positive slope, there is evidence for punctuated evolution in *F*.

Among the 66 language families considered, the *δ*-test was negative for 43 families. We applied Holm-Bonferroni correction for multiple testing to determine significance in the regression analysis. The numerical results are given in Table 5.

**Figure 7:**
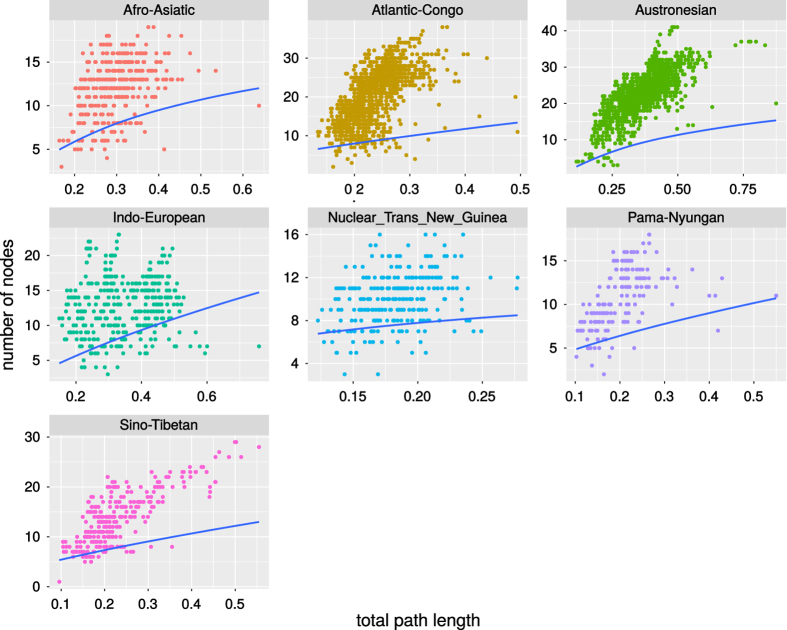
Dependency between total path length and the number of branching nodes for the families with a significantly positive association. Blue lines are regression lines according to phylogenetic generalized least squares, using *δ*-correction.

A significant positive dependency was found for the seven largest language families (Atlantic-Congo, Austronesian, Indo-European, Afro-Asiatic, Sino-Tibetan, Nuclear Trans-New Guinea, Pama-Nyungan). The relationships for these families are visualized in Fig. 7. No family showed a significant negative dependency. This strengthend the conclusion of Atkinson et al.^43^ that languages evolve in punctuational bursts.

## Additional information

**How to cite this article**: Jäger, G. Global-scale phylogenetic linguistic inference from lexical resources. *Sci. Data*. 5:180189 doi: 10.1038/sdata.2018.189 (2018).

**Publisher’s note**: Springer Nature remains neutral with regard to jurisdictional claims in published maps and institutional affiliations.

## Supplementary Material



## Figures and Tables

**Table 1:** Gold standard data used for this study.

**Dataset**	**Source**	**Words**	**Concepts**	**Languages**	**Families**	**Cognate classe**
ABVD	^15^	2,306	34	100	Austronesian	409
Afrasian	^48^	770	39	21	Afro-Asiatic	351
Chinese	^49^	422	20	18	Sino-Tibetan	126
Huon	^50^	441	32	14	Trans-New Guinea	183
IELex	http://ielex.mpi.nl	2,089	40	52	Indo-European	318
Japanese	^51^	387	39	10	Japonic	74
Kadai	^52^	399	40	12	Tai-Kadai	102
Kamasau	^53^	270	36	8	Torricelli	59
Mayan	^6^	1,113	40	30	Mayan	241
Miao-Yao	^52^	206	36	6	Hmong-Mien	69
Mixe-Zoque	^54^	355	39	10	Mixe-Zoque	79
Mon-Khmer	^52^	579	40	16	Austroasiatic	232
ObUgrian	http://starling.rinet.ru	769	39	21	Uralic	68
total		10,106	40	318	13	2,311

**Table 2:** Median Generalized Quartet Distances for Glottolog families.

*Method character type*	Character-based			Distance-based
	Cognate classes	Soundclass-concept	Combined	
total	0.215	0.186	0.173	0.148
small families (n<20)	0.193	0.219	0.219	0.100
medium families (20≤*n*<100)	0.249	0.179	0.171	0.168
large families (*n*≥100)	0.147	0.147	0.102	0.154

**Table 3:** Generalized Quartet Distances to Glottolog expert tree for Glottolog families with≥10 taxa

	character-based				distance-based
family	number of taxa	cognate classes	sound-concept	combined	
Atlantic-Congo	1332	0.147	0.207	0.140	0.178
Austronesian	1259	0.212	0.111	0.110	0.160
Indo-European	367	0.074	0.066	0.062	0.061
Afro-Asiatic	356	0.200	0.147	0.102	0.123
Sino-Tibetan	279	0.075	0.092	0.076	0.154
Nuclear_Trans_New_Guinea	259	0.089	0.167	0.097	0.055
Pama-Nyungan	167	0.230	0.314	0.218	0.214
Austroasiatic	160	0.081	0.050	0.049	0.032
Tai-Kadai	142	0.282	0.261	0.270	0.180
Uto-Aztecan	111	0.327	0.305	0.297	0.242
Mayan	110	0.125	0.085	0.085	0.040
Otomanguean	99	0.219	0.205	0.201	0.195
Mande	76	0.358	0.202	0.163	0.152
Nuclear_Torricelli	63	0.174	0.025	0.028	0.015
Quechuan	62	0.258	0.155	0.150	0.229
Arawakan	61	0.070	0.157	0.116	0.068
Turkic	60	0.333	0.205	0.196	0.125
Timor-Alor-Pantar	59	0.287	0.210	0.224	0.316
Tupian	59	0.249	0.254	0.232	0.223
Central_Sudanic	58	0.185	0.074	0.086	0.096
Nakh-Daghestanian	55	0.062	0.005	0.022	0.013
Nilotic	51	0.249	0.109	0.084	0.028
Dravidian	38	0.160	0.208	0.179	0.187
Hmong-Mien	36	0.229	0.132	0.195	0.195
Ijoid	34	0.032	0.032	0.032	0.032
Pano-Tacanan	33	0.484	0.457	0.438	0.272
Algic	32	0.363	0.372	0.370	0.313
Athapaskan-Eyak-Tlingit	32	0.387	0.187	0.218	0.184
Japonic	32	0.460	0.254	0.154	0.149
Tucanoan	32	0.336	0.243	0.371	0.262
Cariban	30	0.110	0.105	0.106	0.221
Salishan	30	0.143	0.116	0.100	0.000
Uralic	30	0.041	0.019	0.019	0.020
Tungusic	25	0.164	0.177	0.188	0.259
Ta-Ne-Omotic	24	0.129	0.218	0.218	0.093
Chibchan	23	0.384	0.381	0.355	0.362
Sepik	23	0.350	0.059	0.061	0.099
Lakes_Plain	22	0.275	0.181	0.218	0.227
Nuclear-Macro-Je	21	0.485	0.122	0.149	0.151
North_Halmahera	20	0.097	0.204	0.155	0.013
Lower_Sepik-Ramu	19	0.122	0.135	0.000	0.075
Dogon	18	0.063	0.199	0.199	0.100
Angan	17	0.075	0.085	0.085	0.016
Eleman	17	0.145	0.145	0.145	0.145
Siouan	17	0.296	0.028	0.035	0.014
Mixe-Zoque	15	0.091	0.068	0.091	0.091
Gunwinyguan	14	0.251	0.317	0.269	0.211
Mongolic	14	0.283	0.521	0.413	0.351
Sko	14	0.160	0.000	0.000	0.000
Totonacan	14	0.447	0.414	0.414	0.409
Bosavi	13	0.193	0.463	0.248	0.000
Cochimi-Yuman	13	0.301	0.414	0.316	0.316
Khoe-Kwadi	12	0.104	0.377	0.360	0.147
Surmic	12	0.306	0.219	0.219	0.070
Anim	11	0.347	0.000	0.000	0.000
Heibanic	11	0.486	0.489	0.532	0.182
Huitotoan	11	0.485	0.228	0.228	0.295
Kadugli-Krongo	11	0.157	0.324	0.324	0.225
Chocoan	10	0.712	0.225	0.225	0.346
Eskimo-Aleut	10	0.192	0.185	0.185	0.000
Kiwaian	10	0.000	0.571	0.571	0.000
Ndu	10	0.266	0.318	0.318	0.130
Sentanic	10	0.000	0.000	0.000	0.000
Tuu	10	0.333	0.167	0.167	0.167

**Table 4:** Median Generalized Quartet Distances to Glottolog for random samples of languages.

*Method character type*	Character-based			Distance-based
	Cognate classes	Soundclass-concept	Combined	
total	0.187	0.147	0.066	0.130
20≤*n*≤100	0.286	0.210	0.099	0.174
100<*n*≤200	0.226	0.135	0.065	0.115
200<*n*≤300	0.157	0.136	0.063	0.132
300<*n*≤400	0.131	0.132	0.061	0.114

**Table 5:** Test for punctuated language evolution for the families without node density artifact.

Family	Slope	*p*-value	Number of taxa	Significant
Atlantic-Congo	0.003	<1E-14	1332	yes
Austronesian	0.005	<1E-14	1259	yes
Afro-Asiatic	0.008	2E-13	356	yes
Sino-Tibetan	0.005	9E-8	279	yes
Indo-European	0.004	2E-7	367	yes
Nuclear_Trans_New_Guinea	0.003	7E-4	259	yes
Pama-Nyungan	0.005	6E-4	167	yes
Tai-Kadai	0.007	8E-3	142	no
Kiwaian	0.024	9E-3	10	no
Nakh-Daghestanian	0.011	0.01	55	no
Turkic	0.009	0.02	60	no
Quechuan	0.006	0.03	62	no
Siouan	0.004	0.04	17	no
Cariban	0.016	0.05	30	no
Eskimo-Aleut	−0.052	0.05	10	no
Central_Sudanic	0.010	0.07	58	no
Salishan	0.015	0.08	30	no
Chibchan	0.011	0.10	23	no
Ainu	0.013	0.10	22	no
Dravidian	0.008	0.10	38	no
Sko	0.038	0.10	14	no
Uralic	0.017	0.12	30	no
Ndu	0.025	0.14	10	no
Lower_Sepik-Ramu	0.070	0.15	19	no
Japonic	0.010	0.17	32	no
Gunwinyguan	0.027	0.21	14	no
Heibanic	−0.041	0.24	11	no
Khoe-Kwadi	0.015	0.39	12	no
Tungusic	0.011	0.40	25	no
Tucanoan	−0.011	0.45	32	no
Angan	0.018	0.46	17	no
Cochimi-Yuman	0.005	0.47	13	no
Chocoan	−0.027	0.51	10	no
Kadugli-Krongo	−0.006	0.71	11	no
Pano-Tacanan	0.001	0.78	33	no
Tupian	0.001	0.80	59	no
Totonacan	−0.002	0.80	14	no
Ta-Ne-Omotic	−0.003	0.83	24	no
Algic	−0.009	0.86	32	no
Lakes_Plain	−0.002	0.89	22	no
Timor-Alor-Pantar	0.005	0.92	59	no
Bosavi	−0.003	0.99	13	no
Significance is determined via Holm-Bonferroni correction at the significance level of 0.05.				
